# Supplementary material for: Complete realization of energy landscapes and non-equilibrium trapping dynamics in small spin glass and optimization problems
Source: Sci Rep. 2024 Jul 8;14:15675. doi: 10.1038/s41598-024-65493-4 (PMC11231248; doi:10.1038/s41598-024-65493-4)
Supplement: Supplementary file 1 — Supplementary Information. [file 41598_2024_65493_MOESM1_ESM.pdf]

Complete Realization of Energy Landscape and Non-equilibrium Trapping Dynamics in Small Spin Glass and Optimization Problem
Supplementary Information

Ho Fai Po[1,2] and Chi Ho Yeung[1a]
[1]*Department of Science and Environmental Studies, The Education University of Hong Kong, 10 Lo Ping Road, Taipo, Hong Kong.*
[2]*Department of Mathematics, Aston University, B4 7ET, Birmingham, United Kingdom*

Appendix A: Scaling relationships of the number of clusters in CELs in K-Sat problem

In Fig. 2 of the main text, we show that the total number of clusters in CELs in K-Sat problem, $\mathcal{C}$ is proportional to $2^{\gamma N}$, i.e. $\mathcal{C} = A2^{\gamma N}$, for some constant A and exponent γ. Remarkably, as shown in Fig. S1(a), the exponents γ collapse onto a common function of α/K^2 for K-Sat problem with $K = 3, 4, 5$ and 6 and different α. On the other hand, in Fig. S1(b), we show that the ratio $(\mathcal{C}/2^N)^{1/(1-\gamma)}$ collapsed onto a common exponential decay function against N, for $K = 3, 4, 5$ and 6 and different α, implying an extensive number of configurations can be grouped in CELs for clearer illustration. This also implies that the decrease is more extensive with large K, since the satisfiability constraints are less restrictive in clauses with more variables and more configurations can be grouped in clusters because they are more likely to have the same energy. Moreover, this suggests that one can estimate the number of clusters $\mathcal{C}$ for the CELs of a K-Sat problem with arbitrary N, M and K. The same applies for the number of local minima, i.e. $n_{\rm ML}$, which also roughly collapses onto a common function of N as shown in Fig. 2(c) of the main text.

We show the low-energy portion of two more examples of CEL for systems with larger N, namely a spin glass on random regular graph with $N = 20$ and $f_+ = 0.5$ and a 3-Sat problem with $N = 20$ and $\alpha = 4$ respectively in Fig. S2(a) and (b).

Appendix B: More examples on the probability P_g of finding the ground states through FELs, CELs

Starting with a uniform $\vec{P}_0$, we show the sample averaged probability P_g of spin glasses being in the ground state after $t = 10^3$ and 10^5 iteration steps, as well as P_g of 3-Sat problems after $t = 10^4$ and 10^5 iteration steps, as a function of β in Fig. S3(a) and (b) respectively. The probability P_g as a function of time t is shown in the corresponding insets. As we can see, for both systems, the sample averaged P_g first increases with β as expected, but decreases as β further increases. The sample-averaged MCMC simulation results are also in good agreement with theoretical predictions. In

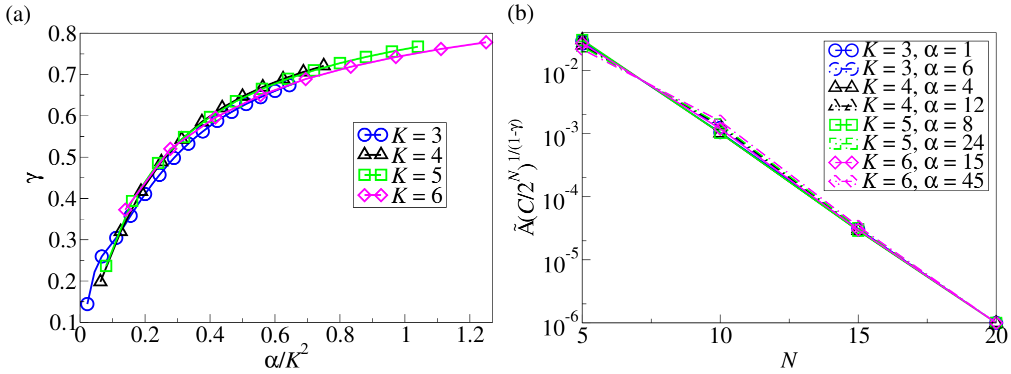

Fig. S1. (a) The exponent γ in $\mathcal{C} \propto 2^{\gamma N}$ as a function of α/K^2 for the K-Sat problem with $K = 3, 4, 5$ and 6. (a) The scaled ratio $\tilde{A}(\mathcal{C}/2^N)^{1/(1-\gamma)}$ between the number of clusters $\mathcal{C}$ in CELs and the corresponding total number of configurations 2^N in FELs as a function of N, where $\tilde{A} = A^{1/(1-\gamma)}$. The results are obtained by averaging over 100 realizations.

[a] chyeung@eduhk.hk

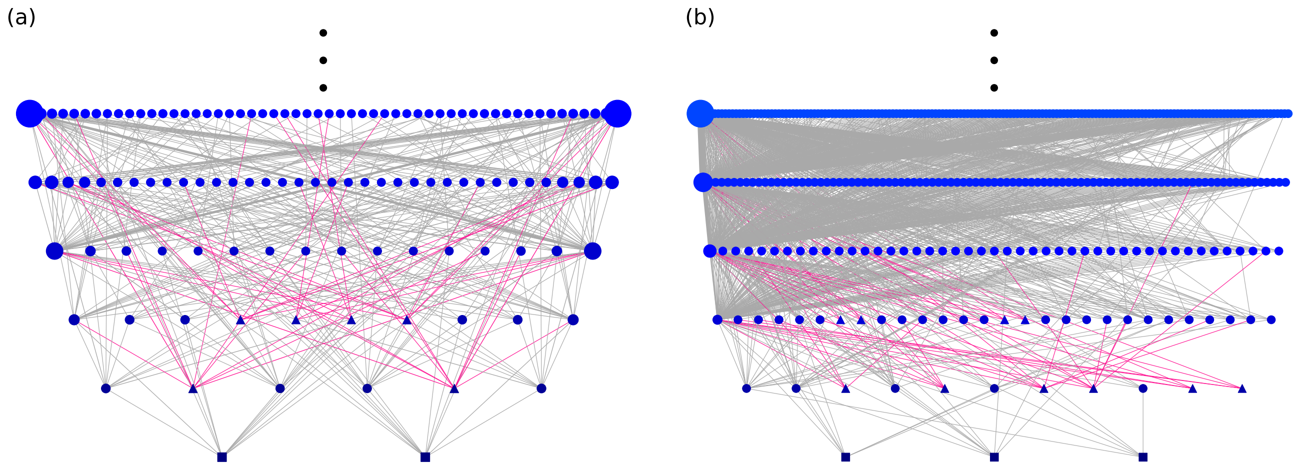

Fig. S2. The low-energy portion of exemplar CELs for an instance of (a) spin glass on random regular graph with $f_+ = 0.5$ and (b) 3-Sat problem with $\alpha = 4$, both with $N = 20$. Global minima and local minima are shown in squares and triangles respectively; node size corresponds to the number of constituent configurations in the clusters; red links correspond to the connections to local minima.

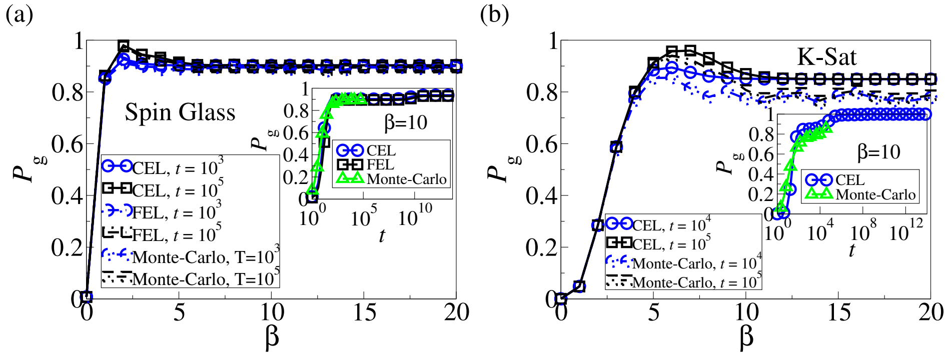

Fig. S3. (a,b) The sample-averaged probability P_g of finding the ground states for spin glass on random regular graphs with $N = 10$ and $f_+ = 0.5$, as well as 3-Sat problems with $N = 15$ and $\alpha = 4$, as a function of inverse-temperature β obtained by Eq. (4) after $t = 10^3$ and 10^5 iterations, compared with simulation results. Insets: P_g as a function of time t. The results are obtained by averaging over 200 realizations.

Fig. S3(a), we also show that the sample-averaged P_g obtained by FELs, which agree well with the results from CELs, implying that CELs capture well the dynamics even after coarse-graining. These results also imply that trapping at local minima is a phenomenon for all instead of particular instances.

Appendix C: PCELs and simplified PCELs

An example of PCEL for a K-Sat problem with $N = 50$ and $\alpha = 4$ is show in Fig. S3(a), obtained by sampling for $T = 10^5$ steps at $\beta_s = 5$ with 10 re-starts. Since the node size of the PCEL is still too large for analysis, and as we have discussed in the main text, we further simplify the PCELs by leaving only nodes on one of the single shortest path between any two minima shown in Fig. S3(b), since we are mainly interested in the glassy behaviors contributed by the local and global minima, .

We then obtain the simplified transition matrix $\tilde{\mathcal{T}}_\beta$ from the simplified PCEL in Fig. S3(b), and compute the probability P_g of the system to be in the ground state after $t = 10^4$ and 10^5 iteration steps as a function of β as in Fig. S5; P_g as a function of time t is shown in the inset. We can see that the simulation results at different β share a similar trend with the theoretical predictions by PCELs, which is obtained by a simple procedure at a single value of β_s.

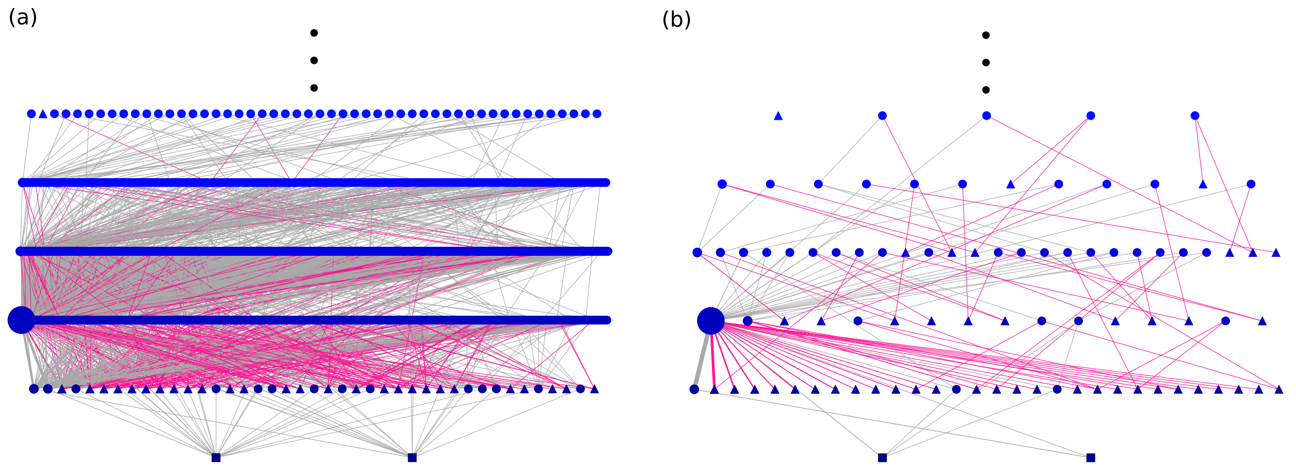

Fig. S4. (a) The low-energy portion of an exemplar PCEL for an instance of 3-Sat problem instance with $N = 50$, as a function of β, sampled for $T = 10^5$ steps at $\beta_s = 5$ with 10 re-starts. (b) The simplified PCEL of (a) by leaving a single shortest path between any two minima only. Global minima and local minima are shown in squares and triangles respectively; node size corresponds to the number of constituent configurations in the clusters; red links correspond to the connections to local minima.

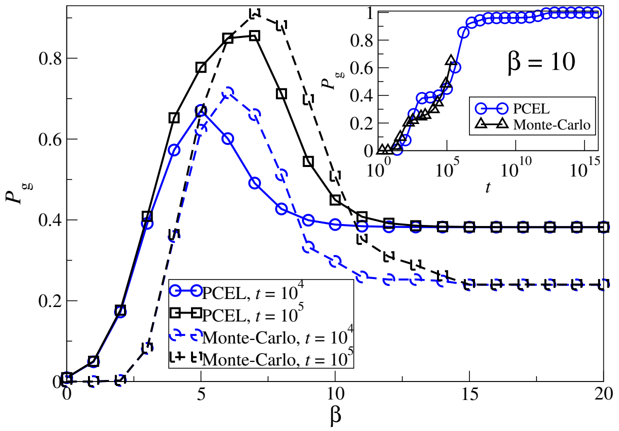

Fig. S5. The sample-averaged probability P_g of finding the ground states of 3-Sat problem instances with $N = 50$, as a function of β, obtained by the transition matrix from PCELs sampled for $T = 10^5$ steps at $\beta_s = 5$ with 10 re-starts, and then by Eq. (4) after $t = 10^4$ and 10^5 iterations, compared with simulation results. Insets: P_g as a function of time t. The results are obtained by averaging 50 realizations.
